# Drug Resistance in People With Viremia on Dolutegravir-based Antiretroviral Therapy in Sub-Saharan Africa: The DTG RESIST Study

**DOI:** 10.1093/cid/ciaf204

**Published:** 2025-05-20

**Authors:** Tom Loosli, Carolyn Bolton Moore, Lydia Buzaalirwa, Helen Byakwaga, İpek Çelikağ, Cleophas Chimbetete, Peter Vanes Ebasone, Jennifer Giandhari, Nuri Han, Jacqueline Huwa, Charles Kasozi, Adolphe Mafoua, Eugène Messou, Albert Minga, Guy Muula, Winnie Muyindike, Arcel Christ Massamba Ndala, Mamatha Sauermann, Aggrey Semeere, Lavanya Singh, Roger D Kouyos, Richard Lessells, Matthias Egger

**Affiliations:** Department of Infectious Diseases and Hospital Epidemiology, University Hospital Zurich, Zurich, Switzerland; Institute of Medical Virology, University of Zurich, Zurich, Switzerland; Centre for Infectious Disease Research in Zambia, Lusaka, Zambia; Center for AIDS Research, School of Medicine, University of Alabama at Birmingham, Birmingham, Alabama, USA; AIDS Healthcare Foundation Uganda Cares, Masaka, Uganda; Faculty of Medicine, Mbarara University of Science and Technology, Mbarara, Uganda; Institute of Social and Preventive Medicine, University of Bern, Bern, Switzerland; Newlands Clinic, Harare, Zimbabwe; Hôpital Jamot, Yaoundé and Regional Hospital, Limbé, Cameroon; KwaZulu-Natal Research Innovation and Sequencing Platform, University of KwaZulu-Natal, Durban, South Africa; Department of Infectious Diseases and Hospital Epidemiology, University Hospital Zurich, Zurich, Switzerland; Institute of Medical Virology, University of Zurich, Zurich, Switzerland; Lighthouse Trust, Lilongwe, Malawi; Public Health Department, Regional Referral Hospital, Masaka, Uganda; Centre de Traitement Ambulatoire, Pointe Noire, Republic of the Congo; Centre de Prise en Charge, de Recherche et de Formation, Abidjan, Côte d’Ivoire; Centre National de Transfusion Sanguine, Abidjan, Côte d’Ivoire; Centre for Infectious Disease Research in Zambia, Lusaka, Zambia; Faculty of Medicine, Mbarara University of Science and Technology, Mbarara, Uganda; Centre de Traitement Ambulatoire, Brazzaville, Republic of the Congo; Institute of Social and Preventive Medicine, University of Bern, Bern, Switzerland; Infectious Diseases Institute, Makerere University, Kampala, Uganda; KwaZulu-Natal Research Innovation and Sequencing Platform, University of KwaZulu-Natal, Durban, South Africa; Department of Infectious Diseases and Hospital Epidemiology, University Hospital Zurich, Zurich, Switzerland; Institute of Medical Virology, University of Zurich, Zurich, Switzerland; KwaZulu-Natal Research Innovation and Sequencing Platform, University of KwaZulu-Natal, Durban, South Africa; Centre for the AIDS Programme of Research in South Africa, Durban, South Africa; Institute of Social and Preventive Medicine, University of Bern, Bern, Switzerland; Centre for Infectious Disease Epidemiology and Research, Faculty of Health Sciences, University of Cape Town, Cape Town, South Africa; Population Health Sciences, Bristol Medical School, University of Bristol, Bristol, United Kingdom

**Keywords:** HIV, dolutegravir, viremia, drug resistance, sub-Saharan Africa

## Abstract

Dolutegravir resistance is an increasing concern. An analysis of the DTG RESIST study found that among 227 integrase sequences from 7 African countries (all non-B subtypes), 59 (26.0%) had at least 1 major drug resistance mutation (primarily G118R and E138A/K/T), with 49 (21.6%) predicted to have high-level resistance to dolutegravir.

Dolutegravir (DTG) is widely used in antiretroviral therapy (ART) programs, particularly in low- and middle-income countries (LMICs) [1]. Acquired DTG resistance is rare in high-income settings [2, 3]. In a study primarily involving participants from European and North American cohorts, the prevalence of major integrase strand transfer inhibitor (INSTI) drug resistance mutations (DRMs) among people with human immunodeficiency virus (HIV, PWH) experiencing viremia on DTG-based ART was 7.2%. High-level resistance to DTG was observed in only 1% of cases [3].

Data on DTG resistance in sub-Saharan Africa are urgently needed, given the region's significant HIV burden and extensive subtype diversity [4]. PWH on ART often experience prolonged periods of viremia in these settings, and access to routine HIV drug resistance testing remains limited [5]. The recent World Health Organization (WHO) drug resistance report highlights surveys from LMICs that show an increasing number of cases of DTG resistance [6].

The DTG RESIST study aims to characterize the prevalence and patterns of DTG resistance among adults and adolescents with virological failure on DTG-based ART across Africa, Asia, and South America [7]. Here, we provide data collected from 7 African countries as of December 2024.

## METHODS

DTG RESIST is nested within the International Epidemiology Databases to Evaluate AIDS (IeDEA) network [8]. The study involves prospective enrollment from routine clinical care at sites in Asia, South America, and sub-Saharan Africa selected to represent diverse settings, programmatic contexts, and non-B HIV subtypes. Enrollment began in June 2022 and will continue until May 2025. Further details are outlined in the study protocol [7]. In brief, the study includes adults and adolescents (aged 10–19 years) with at least 1 routine viral load (VL) >1000 copies/mL while on DTG-based ART. Plasma or dried blood spots (DBS) are collected depending on local storage capacities. Samples are sent to centralized laboratories, and genotypic resistance testing is performed on those with VL >1000 copies/mL.

Here, we present data from participants enrolled between June 2022 and December 2024 at 16 study sites in 7 African countries: Cameroon (n = 2), Côte d’Ivoire (n = 2), Malawi (n = 2), Republic of Congo (n = 2), Uganda (n = 2), Zambia (n = 5), and Zimbabwe (n = 1). Supplementary Table 1 provides further details. Sanger sequencing of integrase (IN) and protease/reverse transcriptase (PR/RT) regions was conducted using the ThermoFisher HIV-1 Genotyping Kit [9]. Sequences were analyzed with the Stanford HIVdb algorithm V9.8 [10], and HIV subtypes were determined using COMET [11] and Rega V3.46 [12].

We report the proportion of individuals with resistance among those with documented viremia (VL >1000 copies/mL) on DTG-based ART, along with the associated mutational patterns.

## RESULTS

The analysis included 488 participants; most were female (N = 284, 58.2%), and 73 (15%) were adolescents. The median duration on DTG-based ART was 2.5 years (interquartile range [IQR], 1.6–3.4). Most (n = 447, 91.6%) were on a regimen that contained DTG with 2 Nucleos(t)ide Reverse Transcriptase Inhibitors (NRTIs), with 336 of 447 (75.2%) receiving tenofovir/lamivudine/dolutegravir (TLD). Most participants (n = 421, 86.3%) had prior exposure to at least 1 ART regimen, while 67 (13.7%) were treatment-naive when they started DTG. Previous exposure to raltegravir was rare (n = 9, 1.8%). Most participants (n = 415, 85%) had 2 or more consecutive VL measurements >1000 copies/mL before enrollment. The median time between the most recent routine VL >1000 copies/mL and enrollment was 36 days (IQR, 22–69). Supplementary Tables 2 and 3 provide further details.

**Figure 1.**
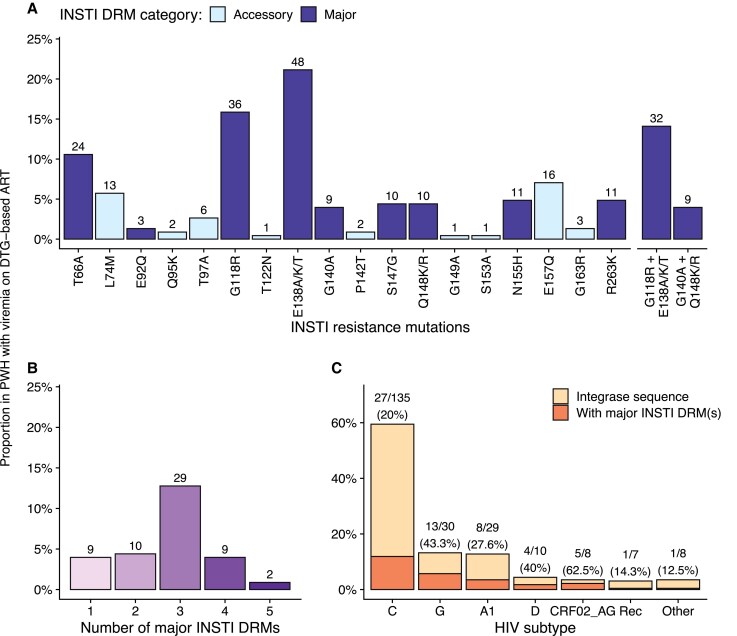
INSTI DRMs in people with non-B HIV-1 experiencing virological failure on DTG-based ART in 7 African countries. *A*, INSTI DRMs among individuals with 1 or more major INSTI DRMs and, among the latter, the most frequent combinations of INSTI DRMs. *B*, Number of major INSTI DRMs. (*C*) HIV subtypes in those with integrase sequences and proportion with 1 or more major INSTI DRMs. Subtypes labeled as other include H (N = 3), unknown (N = 3), J (N = 1), and F (05_DF) (N = 1). Abbreviations: ART, antiretroviral therapy; DRM, drug resistance mutation; DTG, dolutegravir; HIV, human immunodeficiency virus; INSTI, integrase strand transfer inhibitor; PWH, people with human immunodeficiency virus.

Of the 488 samples (420 plasma, 68 DBS samples), 234 (48%) had a VL >1000 copies/mL and underwent sequencing. IN sequencing was successful for 227 of 234 participants (97.0%), and PR/RT sequencing was successful for 217 of the 227 with IN sequences (95.6%). Overall, 59 of 227 (26.0%) participants had major INSTI DRMs (Figure 1*A*). The most frequent major INSTI DRMs were G118R (n = 36) and E138A/K/T (n = 48), occurring together in 32 study participants (Figure 1*A*). R263K was less common, as were G140A and Q148K/R. Most participants (50 of 59, 84.7%) had 2 or more major INSTI DRMs (Figure 1*B*). The most frequent HIV subtypes were C (n = 135, 62.2%), G (n = 30, 13.8%), and A1 (n = 29, 13.4%); the prevalence of major DRMs varied across subtypes (Figure 1*C*).

All 59 cases with major INSTI DRMs were treatment-experienced, including 7 of 41 (17.1%) adolescents with major INSTI DRMs. Median duration on DTG-based ART was 2.8 years (IQR, 2.1–3.4) and 2.4 years (IQR, 1.6–3.4) for participants with and without major INSTI DRMs, respectively. High-level resistance to DTG was predicted in 49 of 227 (21.6%) participants; across countries, the prevalence ranged from 11.4% to 40.9% (Supplementary Table 4). Among the 67 participants who were ART-naive when they started DTG, 21 had VL >1000 copies/mL, none had major INSTI DRMs, and 4 had accessory DRMs (Q95K, H51Y, E157Q, and S153A).

PR/RT sequencing was successful for 57 of the 59 participants with major INSTI DRMs; 55 (96.5%) harbored M184V. In contrast, K65R was rare, occurring in 5 (8.8%) participants. Thymidine analogue mutations (TAMs) were detected in 41 of 57 (71.9%) participants, with 21 of 41 (51.2%) having 3 or more TAMs. Of the 41 participants with TAMs and major INSTI DRMs, 27 (65.9%) were on TLD at enrollment. Intermediate or high-level tenofovir resistance was identified in 18 of 57 (31.6%) participants with major INSTI DRMs. Among 158 participants susceptible to DTG (with PR/RT sequencing available), M184V was found in 17 (10.8%), while 11 (7.0%) had at least 1 TAM. Intermediate or high-level tenofovir resistance was observed in 3.8% of these cases. Protease inhibitor (PI) resistance was rare. Major PI DRMs were detected in 8 of 57 (14.0%) participants with major INSTI DRMs. All participants with PI DRMs had predicted susceptibility to darunavir.

## DISCUSSION

In this analysis of African DTG RESIST data, high-level DTG resistance was detected in about 1 in 5 people with non-B HIV-1 and experiencing virological failure on DTG-based ART. The most common mutations were G118R and E138A/K/T, frequently occurring in combination. All individuals with major INSTI DRMs were treatment-experienced, and the majority also had NRTI resistance. Although few participants were on PIs at the time of resistance testing, the rarity of PI resistance mutations suggests that PIs may still be an option for those experiencing virologic failure on DTG-based ART.

Our findings align with the findings presented in a recent WHO report on DTG resistance. The report estimated that the prevalence of DTG resistance among individuals with viremia on DTG-based ART in sub-Saharan Africa ranged from 3.9% to 19.6% [6]. The country-specific DTG resistance prevalence observed in our study falls within this range, but numbers are small, and country differences must be interpreted cautiously. The differences observed may reflect variations in the calendar time of the DTG rollout in different countries, as well as different inclusion criteria, implementation practices, or study populations. Nevertheless, a common finding across studies is the rising prevalence of DTG resistance among individuals experiencing virological failure on DTG-based ART [6].

The DTG resistance patterns observed in sub-Saharan Africa in this study and the studies covered in the WHO report [6] contrast with analyses from high-income settings [2, 3]. Among individuals with virological failure on DTG-based ART, high-level DTG resistance was approximately 20 times more frequent in sub-Saharan Africa than in Europe or North America [3]. Additionally, the mutational pathway in sub-Saharan Africa primarily involved G118R in combination with E138A/K/T, while in the European and North American cohorts, the predominant pathway was R263K. DTG resistance levels also differed. In high-income settings, potential low- or intermediate-level resistance dominated, and high-level resistance was rare [3]. By contrast, in sub-Saharan Africa, most people with resistance had high-level DTG resistance, often due to multiple major INSTI DRMs. This situation may reflect the individualized care in high-income countries, with frequent VL monitoring and genotypic resistance testing performed earlier during virological failure than in ART programs in resource-limited African settings. The differences in HIV-1 subtypes could also play a role [3]. Of note, those with major INSTI DRMs were treatment-experienced individuals in both settings. Understanding these differences is crucial for developing strategies for preventing drug resistance.

The strengths of this study include the large number of sites and ART programs in 7 countries that participate in IeDEA, a long-standing collaboration of routine ART programs in sub-Saharan Africa [8]. Of note, the study sites reflect different approaches to switching PWH to DTG-based ART, including sites where switching was irrespective of viral load and sites where switching depended on a suppressed viral load in the past year [13]. The IeDEA collaboration collects a wide range of longitudinal data, allowing future analyses of clinical outcomes and the durability and effectiveness of DTG-based ART regimens over time. Our study also has several limitations. Currently, the data on the duration of viremia during DTG-based ART and prior ART regimens are incomplete; however, linking study data to the broader IeDEA cohort will allow more detailed analyses of the factors that contribute to resistance. Samples with detectable viral loads below 1000 copies/mL were not sequenced. We may, therefore, have missed early emergent resistance mutations. Also, drug levels were not measured, which prevented insights into the relationship between adherence and resistance. Finally, we used the Stanford HIVdb algorithm but acknowledge that other systems may classify mutations differently and that discriminating between major and accessory (or major and minor) is not always clear-cut. For example, the E138A/K/T mutation is not considered a major mutation in the drug mutation chart of the International AIDS Society (IAS)-USA.

In conclusion, DTG remains a cornerstone of HIV treatment, achieving high rates of viral suppression in most PWH. DTG resistance among those newly initiating the drug remains low. However, this interim analysis of data from the sub-Saharan African sites of DTG RESIST shows that 1 in 5 individuals on failing DTG-based ART harbored high-level DTG resistance, most commonly through the G118R and E138A/K/T mutational pathways. Safeguarding the efficacy of INSTI-based ART in LMICs will require proactive measures to address the emergence of DTG resistance, ensuring sustained success in HIV treatment.

## Supplementary Material

ciaf204_Supplementary_Data

## Supplementary Data

Supplementary materials are available at *Clinical Infectious Diseases* online. Consisting of data provided by the authors to benefit the reader, the posted materials are not copyedited and are the sole responsibility of the authors, so questions or comments should be addressed to the corresponding author.

## Notes

***Author Contributions*.** T. L., R. D. K., R. L., and M. E. led the conceptualization and writing of the first draft. All authors provided critical intellectual content for revising the draft manuscript. All authors had full access to the information described in the manuscript.

***Acknowledgments*.** We thank the patients and healthcare providers who participate in the DTG RESIST study of International Epidemiology Databases to Evaluate AIDS.

***Data sharing***. The sequences have been uploaded to GenBank under accession numbers PV008448-PV008641, PV407014-PV407203, and PV419497-PV419583.

***Financial support.*** This work was supported by the National Institute of Allergy and Infectious Diseases (NIAID; award R01AI152772 to M. E.) and the Swiss National Science Foundation (award 32FP30_207285 to M. E. and award 324730_207957 to R. D. K.).
