# Supplementary material for: Drug Resistance in People With Viremia on Dolutegravir-based Antiretroviral Therapy in Sub-Saharan Africa: The DTG RESIST Study
Source: Clin Infect Dis. 2025 May 20;81(4):e128–31. doi: 10.1093/cid/ciaf204 (PMC12596406; doi:10.1093/cid/ciaf204)
Supplement: ciaf204_Supplementary_Data [file ciaf204_supplementary_data.docx]

# Supplement to:

Drug resistance in people with viremia on dolutegravir-based ART in sub-Saharan Africa: the DTG RESIST study. Tom Loosli et al. for the DTG RESIST Study Group

**DTG RESIST study group:**

*Argentina: Administration (Cinthia Sapienza); Clinical Staff (Camila Valeriano, Carolina Perez, Estefania Rodriguez, Florencia Cahn, Maria Victoria Iannantuono, Patricia Patterson, and Victoria Viera); Co-PI (Carina César); Data Management (Nicolás Doudtchitzky); Laboratory Staff (Ana Gun and Mariana Ferrari); PI (Pedro Cahn); Study coordination (Horacio Beylis and Lara Vladimirsky)*

*Brazil: Administration (Beatriz Leonardo, Claudia Silva, Gabriel Silva, Manoel Filho, Marcella Barboza, Sue Lima, Tania Krstic, and Ubiraçan Rufino); Co-PI (Livia Ferreira); Data Management (Alexandre Souza, Ana Carolina Figueiredo, Ana Claudia Silva, Flavia Lessa, Luiz Camacho, Ronaldo Moreira, and Thadeu Pinheiro); Laboratory Staff (Ellen Gomes, Flavia Gomes, Liriell Cordeiro, Michelli Gonçalves, Sandro Costa, and Soraia Moura); PI (Sandra Cardoso); Research (Barbara Viggiani, Daniel Arabe, and Davila Silva)*

*Burkina Faso: Administration (Sidia Arlette Sanou); Clinical Staff (Sanata Koala, Sidbéwindin Richard Ramde, and Stéphane Sanou); Data Management (Gbolo Pooda); Laboratory Staff (Abdoul-Salam Ouedraogo, Maxime Damolga, and Yacouba Sawadogo); PI (G.e. Armel Poda); Psychosocial counselor (Micheline Sanou)*

*Cambodia: Clinical Staff (Mengsomanythd Chhay, Narom Prak, Seila Pech, Sophea Heng, and Sreypov Heam); Co-PI (Mengsomanythd Chhay); Data Management (Chanthy Pov, Sopanha Pich, and Sophea Heng); Laboratory Staff (Chandara Mom and Sopanha Pich); PI (Vohith Khol)*

*Cameroon: Administration (Clarisse Lengouh); Clinical Staff (Djenabou Amadou, Eric Ngassam, Ivon Nchang, and Phyllis Fon); Data Management (Mabou Gabriel and Marc Lionel Ngamani); IeDEA Coordination (Jordanne Ching and Judith Nasah); PI (Anastase Dzudie); Study coordination (Peter Vanes Ebasone)*

*Cote d’Ivoire CNTS: Clinical Staff (Arlette Kouamé Epouse Kouadio, Bades Isidore Bohouo, Hosihiri Lambert Dohoun, and You Linda Niagne Epouse Téhé); Co-PI (Kouadio Stéphane N’goran); Data Management (Konan Raoul Kouakou); Laboratory Staff (Mathias Balie and Vincent Camille Sablin); PI (Kla Albert Minga)*

*Cote d’Ivoire CePREF: Clinical Staff (Amah Cécile Tchehy and Jeannot Goli); Co-PI (Anzian Amani); Data Management (Emma Nadège Kokogny and Issouf Koffi Ladji); Laboratory Staff (Sammuel Assande); PI (Eugène Messou); Research (Karidiatou Diallo)*

*Durban Lab: Co-PI (Jennifer Giandhari and Tulio De Oliveira); Data Management (Bertha Baye and Hastings Musopole); Laboratory Staff (Lavanya Singh, Nonkululeko Avril Mbatha, Samukelisiwe Pretty Khathi, Shirelle Naidoo, Sureshnee Pillay, and Xolani Hilorious Zulu); PI (Richard John Lessells)*

*India: Administration (Beulah Balakrishnan); Clinical Staff (Dr.keerthana Priya Thamayanthi Shankar, Esther Mony, and Ramya Murugan); Co-PI (Dr.poongulali Selvamuthu); Data Management (Blessy Bobby, Jacinth Sugumar, Rajesh Elangovan, Sasi Jothiramalingam, and Shilpa Jose); Laboratory Staff (Dr. Priya Kannian, Gracemary Arul, Harika Paila, Mahanathi Pasuvaraj, Shalini Loganathan, and Sivaranajani Durairaj); PI (Dr. Kumarasamy Nagalingeswaran) Kenya: Clinical Staff (Cosmas Apaka and Julius Cheruiyot); Co-PI (Shamim M. Ali and Suzanne Goodrich); PI (Kara Wools-Kaloustian and Lameck Diero)*

*Malawi: Administration (Joseph Chintedza); Clinical Staff (Erick Mtemang’ombe, Jessie Hau, Kelvin Rambiki, and William Maliko); Co-PI (Jacqueline Huwa, Rose Nyirenda, and Wilson Bilaal); Data Management (Blessings Mwandira, Geldert Chiwaya, and Pachawo Bisani); Laboratory Staff (Enock Khunju, Rafiq Maluwa, and Shameem Buleya)*

*Mexico Lab: Data Management (Claudia Garcia-Morales); Laboratory Staff (Margarita Matias-Florentino); PI (Santiago Avila-Rios) Rwanda: Administration (Marie Gertrude Bahire Rutwaza); Clinical Staff (Fabienne Shumbusho, Francine Umwiza, Samuel Munyentwari, and Verene Mukankurunziza); Co-PI (Marcel Yotebieng); Data Management (Benjamin Muhoza); Laboratory Staff (Faustin Kanyabwisha); PI (Gad Murenzi)*

*Thailand HIVNAT: Co-PI (Napon Hiranburana); Data Management (Chuleeporn Wongvoranet and Penpanat Toomcharoen); Laboratory Staff (Sasiwimol Ubolyam); PI (Anchalee Avihingsanon)*

*Thailand Lab: Laboratory Staff (Suwanna Mekprasan); PI (Sunee Sirivichayakul) Thailand Ramathibodi: Clinical Staff (Laor Nakgul and Nutaporn Sanmeema); Co-PI (Asoc.angsana Phuphuakrat); Data Management (Laor Nakgul and Nutaporn Sanmeema); Laboratory Staff (Laor Nakgul and Nutaporn Sanmeema); PI (Prof.sasisopin Kiertiburanakul)*

*Uganda Masaka: Clinical Staff (Aloysius Ssabayinda, Dennis Madanda, Monica Nakirya, Samuel Katungi, Stella Nabunnya, and Wilson Kazoora); Data Management (Matthew Ssemakadde and Phoebe Mutenyo); Laboratory Staff (Moses Asiimwe); PI (Charles Kasozi and Lydia Buzaalirwa)*

*Uganda Mbarara: Administration (Bronia Mwiine); Clinical Staff (Alexis Byaruhanga, Bob Ssekyanzi, Caroline Kusingura, Lillian Ayesiga, and Sarah Namwanje); Co-PI (Helen Byakwaga); Data Management (David Muhumuza); Laboratory Staff (Yona Mbalibulha); PI (Winnie Muyindike)*

*Zambia: Clinical Staff (Alice Miyanda, Aretha Mumba, Fiona Mureithi, Kenan Simumba, and Vivian Tonga); Co-PI (Guy K. Muula); Data Management (Caroline Chileshe, Esau Banda, Ethel Muyanga, Jackson Daka, Josephine Mboozi, Suwilanji Nalungwe, and Sydney Kamiji); Laboratory Staff (Chenge Mukonde, Choolwe Bwalya, Paul Pandala, and Tabiso Mubiana); PI (Carolyn Bolton); Peer educator (Pamela Kabombo and Thandiwe Phiri)*

*Zimbabwe: Clinical Staff (Paddington Marume, Varaidzo Kachingwe, and Wilson Marikopo); Data Management (Ardele Mandiriri, Maureen Wellington, Sydney Malunga, and Varaidzo Moyo); Laboratory Staff (Dakarayi Magumise and Ratidzai Katsidzira); PI (Cleophas Chimbetete)*

Supplemental table 1: Number of study participants per country and site included in this analysis

| Country | Study sites | Participants |
| --- | --- | --- |
| Cameroon (First enrollment: Jun 2023) | Hopital Jamot | 18 |
|  | Limbe Regional Hospital | 16 |
| Congo (First enrollment: Feb 2023) | Centre de Traitement Ambulatoire Pointe-Noire | 29 |
|  | Centre de Traitement Ambulatoire, Brazzaville | 34 |
| Cote d'Ivoire (First enrollment: Oct 2022) | ACONDA Centre de Prise en Charge et de Formation - CePReF | 11 |
|  | Centre médical de suivi des donneurs de sang, CNTS | 31 |
| Malawi (First enrollment: Jun 2023) | Lighthouse | 15 |
|  | Martin Preuss Centre - MPC | 32 |
| Uganda (First enrollment: Oct 2022) | Masaka | 53 |
|  | Mbarara | 54 |
| Zambia (First enrollment: Jun 2022) | Zambia-CIDRZ-Kalingalinga | 19 |
|  | Zambia-CIDRZ-Kanyama | 21 |
|  | Zambia-CIDRZ-Matero | 23 |
|  | Zambia-CIDRZ-UTH Adult | 64 |
|  | Zambia-CIDRZ-UTH Pediatric | 25 |
| Zimbabwe (First enrollment: May 2023) | Newlands | 43 |
| Total | - | 488 |

Supplemental table 2: Characteristics of study participants.

|  | Integrase sequenced | No Integrase sequence | Overall |
| --- | --- | --- | --- |
|  | (N=227) | (N=261) | (N=488) |
| Sex |  |  |  |
| Female | 142 (62.6%) | 142 (54.4%) | 284 (58.2%) |
| Male | 85 (37.4%) | 119 (45.6%) | 204 (41.8%) |
| Age group |  |  |  |
| Adult (>19 years) | 186 (81.9%) | 229 (87.7%) | 415 (85.0%) |
| Adolescent (10-19 years) | 41 (18.1%) | 32 (12.3%) | 73 (15.0%) |
| ART regimen at enrollment* |  |  |  |
| DTG+3TC+TDF | 146 (64.3%) | 190 (72.8%) | 336 (68.9%) |
| DTG+3TC+AZT | 33 (14.5%) | 24 (9.2%) | 57 (11.7%) |
| DTG+3TC+ABC | 16 (7.0%) | 18 (6.9%) | 34 (7.0%) |
| DTG+3TC+DRV+RTV+TDF | 10 (4.4%) | 10 (3.8%) | 20 (4.1%) |
| DTG+FTC+TAF | 6 (2.6%) | 7 (2.7%) | 13 (2.7%) |
| DTG+PI+NNRTI±NRTI | 6 (2.6%) | 1 (0.4%) | 7 (1.4%) |
| DTG+PI±NRTI | 5 (2.2%) | 7 (2.7%) | 12 (2.5%) |
| DTG+2NRTI | 4 (1.8%) | 3 (1.1%) | 7 (1.4%) |
| Unknown | 1 (0.4%) | 1 (0.4%) | 2 (0.4%) |
| Virus loads before for enrollment |  |  |  |
| Two VL >1000 cp/ml | 209 (92.1%) | 207 (79.3%) | 416 (85.2%) |
| One VL >1000 cp/ml | 18 (7.9%) | 54 (20.7%) | 72 (14.8%) |
| Sample type |  |  |  |
| Plasma | 203 (89.4%) | 217 (83.1%) | 420 (86.1%) |
| DBS | 24 (10.6%) | 44 (16.9%) | 68 (13.9%) |

* The ART regimen occurring less than 10 times were grouped by drug class combinations. The full list of regimens is as follows: DTG+2NRTI: 3TC+DTG+TAF (N=3) and DTG+FTC+TDF (N=4), DTG+PI+NNRTI±NRTI: 3TC+DRV+DTG+ETR+RTV+TDF (N=5), DRV+DTG+ETR+RTV+TDF (N=1), and DRV+DTG+ETR+TDF (N=1), DTG+PI±NRTI: 3TC+ATV+AZT+DTG+RTV (N=1), 3TC+ATV+DTG+TAF (N=1), 3TC+ATV+DTG+TDF (N=2), 3TC+AZT+DRV+DTG+RTV (N=1), 3TC+DRV+DTG+TDF (N=2), 3TC+DTG+LPV+RTV+TDF (N=1), DRV+DTG+FTC+RTV+TAF (N=1), DRV+DTG+FTC+RTV+TDF (N=1), DRV+DTG+RTV (N=1), and DTG+FTC+LPV+RTV+TAF (N=1), and Regimen needs confirmation: 3TC+DTG+EFV+RTV+TDF (N=1) and AZT+DTG+TDF+XTC (N=1)

Prior exposure to first-generation integrase strand transfer inhibitors (INSTIs) could lead to distinct INSTI drug resistance mutation (DRM) patterns. Among the nine participants with documented prior exposure to raltegravir, four had study samples with viral loads >1000 copies/mL. Although the sample size is small and limits broader conclusions, it is notable that three of these four participants had major INSTI DRMs, and that these did not involve the G118R or R263K resistance pathways. See table below.

Supplemental table 3: Viral load and INSTI DRMS among nine participants with documented prior exposure to raltegravir.

| Virus load in study sample | Major INSTI DRMs | Accessory INSTI DRMs |
| --- | --- | --- |
| < 1'000 cp/ml | *-* | *-* |
| < 1'000 cp/ml | *-* | *-* |
| < 1'000 cp/ml | *-* | *-* |
| < 1'000 cp/ml | *-* | *-* |
| < 1'000 cp/ml | *-* | *-* |
| >= 1'000 cp/ml | E92Q, E138K, S147G, N155H | E157Q |
| >= 1'000 cp/ml | None | None |
| >= 1'000 cp/ml | E138K, G140A, Q148K | None |
| >= 1'000 cp/ml | E138K, G140A, S147G, Q148K | None |

Supplemental table 4: DTG resistance levels by countries

| Country | Total | Susceptible | Potential-low | Intermediate | High |
| --- | --- | --- | --- | --- | --- |
| Cameroon | 9 | 6 |  | 1 | 2 (22.2%) |
| Côte d’Ivoire | 22 | 10 | 1 | 2 | 9 (40.9%) |
| Malawi | 35 | 29 | 2 |  | 4 (11.4%) |
| Republic of Congo | 32 | 22 |  |  | 10 (31.2%) |
| Uganda | 30 | 23 |  | 1 | 6 (20.0%) |
| Zambia | 76 | 59 | 1 | 4 | 12 (15.8%) |
| Zimbabwe | 23 | 17 |  |  | 6 (26.1%) |
| Total | 227 | 166 | 4 | 8 | 49 (21.6%) |
